# Distinct non-ischemic myocardial late gadolinium enhancement lesions in patients with type 2 diabetes

**DOI:** 10.1186/s12933-020-01160-y

**Published:** 2020-10-22

**Authors:** Annemie Stege Bojer, Martin Heyn Sørensen, Niels Vejlstrup, Jens P. Goetze, Peter Gæde, Per Lav Madsen

**Affiliations:** 1 Department of Cardiology and Endocrinology, Slagelse Hospital, Ingemannsvej 32, 4200 Slagelse, Region Zealand Denmark; 2 Faculty of Health Sciences, Institute of Regional Health Research, University of Southern Denmark, Odense, Denmark; 3 Department of Cardiology, Rigshospitalet, Copenhagen, Denmark; 4 Department of Clinical Biochemistry, Rigshospitalet, Copenhagen, Denmark; 5 Department of Cardiology, Copenhagen University Hospital Herlev-Gentofte, Capital Region of Denmark, Herlev-Gentofte, Denmark; 6 Department of Clinical Medicine, University of Copenhagen, Copenhagen, Denmark

**Keywords:** Diabetes type 2, Cardiovascular magnetic resonance imaging (MRI), Risk factors, Late gadolinium enhancement, Diabetes complications

## Abstract

**Background:**

Cardiovascular magnetic resonance imaging (CMR) have described localised non-ischemic late gadolinium enhancement (LGE) lesions of prognostic importance in various non-ischemic cardiomyopathies. Ischemic LGE lesions are prevalent in diabetes (DM), but non-ischemic LGE lesions have not previously been described or systematically studied in DM.

**Methods:**

296 patients with type 2 DM (T2DM) and 25 sex-matched control subjects underwent echocardiography and CMR including adenosine-stress perfusion, T_1_-mapping and LGE.

**Results:**

264 patients and all control subjects completed the CMR protocol. 78.4% of patients with T2DM had no LGE lesions; 11.0% had ischemic LGE lesions only; 9.5% had non-ischemic LGE lesions only; and 1.1% had both one ischemic and one non-ischemic lesion. The non-ischemic LGE lesions were situated mid-myocardial in the basal lateral or the basal inferolateral part of the left ventricle and the affected segments showed normal to high wall thickness and normal contraction. Patients with non-ischemic LGE lesions in comparison with patients without LGE lesions had increased myocardial mass (150 ± 34 vs. 133 ± 33 g, P = 0.02), average E/e’(9.9 IQR8.7–12.6 vs. 8.8 IQR7.4–10.7, P = 0.04), left atrial maximal volume (102 IQR84.6–115.2 vs. 91 IQR75.2–100.0 mL, P = 0.049), NT-proBNP (8.9 IQR5.9–19.7 vs. 5.9 IQR5.9–10.1 µmol/L, P = 0.02) and high-sensitive troponin (15.6 IQR13.0–26.1 vs. 13.0 IQR13.0–14.6 ng/L, P = 0.007) and a higher prevalence of retinopathy (48 vs. 25%, P = 0.009) and autonomic neuropathy (52 vs. 30.5%, P = 0.005).

**Conclusion:**

A specific LGE pattern with lesions in the basal lateral or the basal inferolateral part of the left ventricle was found in patients with type 2 diabetes.

*Trial registration* https://www.clinicaltrials.gov. Unique identifier: NCT02684331.

## Background

For decades it has been known that angiopathy, as seen in patients with diabetes (DM) differs from what is seen in other patients [1], and the cardiomyopathy associated with DM has increasingly been recognised to be a specific entity. People with DM, compared to people without, have a three-fold increased risk of death from cardiovascular causes [2] and a two- (men) to five-fold (women) increased relative risk for heart failure [3]. The increased risk for heart disease not only on ischemic but also on a non-ischemic background has been confirmed extensively [4]. However, the cardiac phenotype in DM and hence possible points for intervention is still not well characterized.

In DM, even without significant coronary atheromatosis, altered cellular metabolism and hyperglycaemia result in oxidative stress, microvascular angiopathy, and diffuse interstitial myocardial fibrosis [5]. With echocardiography and cardiovascular magnetic resonance imaging (CMR), it has been shown that the cardiac phenotype of DM is associated with stiffening of the left ventricle (LV) with increased myocardial extracellular volume (ECV) and prevalent signs of diastolic dysfunction [6, 7]. In other forms of non-ischemic cardiomyopathy, areas of late gadolinium enhancement (LGE) not explained by ischemic heart disease have been described, but such have not before been described or indeed systematically studied in patients with DM. CMR is the non-invasive reference standard for cardiac volumes and myocardial mass and allows for quantification of ECV, myocardial perfusion and for localised LGE lesions of ischemic as well as non-ischemic origin [8].

It is important to determine if and to what degree non-ischemic LGE lesions exist in patients with DM and to understand their relations to not only risk factors but also variables of known importance for cardiac function and prognosis. In a larger cross-sectional CMR study of patients with type 2 DM (T2DM), we therefore systematically characterized all LGE lesions and their associations with myocardial perfusion, myocardial structure, signs of diastolic dysfunction, and association to common complications of DM.

### Methods

## Study design and population

T2DM patients were recruited from the Department of Endocrinology at Næstved, Ringsted, and Slagelse Hospitals in Denmark. After written informed consent, patients were included between January 2016 and August 2019. The study complies with the Declaration of Helsinki and was approved by The Zealand Ethics Committee (SJ-490). T2DM patients aged 18–80 years and diagnosed with T2DM for at least three months prior to inclusion were eligible for inclusion. Exclusion criteria were persistent or permanent atrial fibrillation at the time of echo or CMR, eGFR < 30 mL/min/1.73 m^2^, claustrophobia or other contraindications to CMR. Sex-matched normal subjects served as a control group, all without T2DM (HbA1c < 40 mmol/mol). Antihypertensive and statin therapy were allowed in the control group.

Patients underwent full medical history, assessment of blood pressure, electrocardiogram (ECG), and echocardiography. Blood and urinary samples were collected prior to the CMR scan, which in general was performed within 14 days of other assessments. History of retinopathy (latest fundoscopy), peripheral neuropathy (latest chiropodist report), ischemic heart disease, hypertension (BP > 140/90 mmHg), and medication were obtained from the patient journal. An ECG obtained during five cycles of in- and expiration was performed to allow for beat-to-beat variation being analysed. Orthostatic blood pressure measurements were obtained ½, 1½, 3, 5, and 7 min after acutely standing up [9]. Patients with an orthostatic decrease in systolic blood pressure > 25 mmHg in any of the measurements or a beat-to-beat variation ≤ 6 bpm, or both, were diagnosed with impaired autonomic neural function [9, 10]. Albuminuria was defined as a urinary albumin/creatinine-ratio of > 30 mg/g.

### Echocardiography protocol and data analysis

Standard 2D echocardiography was performed using a Vivid S5 probe on a GE healthcare Vivid E9 cardiovascular ultrasound system. The GE EchoPAC workstation was used for analysis. Peak early mitral inflow (E) was measured in the 4-chamber view using pulsed-wave Doppler with the sample volume placed between the tips of the mitral valve leaflets. Also, in the 4-chamber view, the early diastolic myocardial velocity (e′) was measured using pulsed-wave tissue velocity Doppler with the sample volume placed in the septal and lateral mitral annulus.

### Cardiac MRI protocol

CMR studies were performed on a 1.5 T scanner (Siemens Avanto, n = 247 patients and all healthy controls or GE healthcare Optima 450w, n = 19 patients) with patients in a supine position on the back using a surface- and spine cardiac coil with ECG gating. Cardiac short-axis steady-state free precession cine sequences were acquired during end-expiratory breath-holds in 25 phases (covering the whole heart; slice thickness 8 mm, no gap) and in 2-, 3- and 4-chamber views. Sequence settings were TE 1.16–1.25 ms, TR 46.24–49.98 ms, Matrix 210 × 208, FoV 258 × 320–485 × 481. Myocardial perfusion images were obtained at the basal, mid-ventricular and apical cardiac short-axis level during rest and pharmacological stress (adenosine 140 µg/kg/min) using an iv. dose of 0.075 mmol/kg of gadobutrol (Gadovist®, Bayer AG, Germany) for each sequence as previously described [11]. LGE imaging was performed using a phase-sensitive segmentation gradient echo inversion recovery sequence following selection of the appropriate TI time based on a Look-Locker sequence. Approximately 5 min after the last contrast dose, a short axis LGE stack of the LV was performed (slice thickness 8 mm, no gap, TE 3.16–3.20 ms, TR 537-748 ms, Matrix 224 × 256–256 × 256, FoV 318 × 340–443 × 458). This was followed by LGE images in the 2-, 3- and 4-chamber views. A trained physician reviewed LGE images during the scan and any LGE lesions seen in the short axis images were confirmed from perpendicular slices through the lesion in question. Additionally, native and post-contrast T_1_-mapping were performed as previously described [11] (not performed in the 19 patients scanned with the GE healthcare Optima 450w).

### Cardiac MRI analysis

CMR images were analysed using cvi42^©^ (Circle Cardiovascular Imaging Inc., Calgary Canada, v. 5.5.1). LV volumes and mass were determined by semi-automatic tracing of the endo- and epicardial contours in end-diastolic and end-systolic phases. Papillary muscles were excluded. LA maximum volume was measured by manual tracing in the LV end-systolic frame. For all CMR parameters we chose to report both the absolute values and indexed to body surface area because the majority of our patients were severe obese, which could bias the indexed results. A LGE lesion was considered to be present if seen in at least two perpendicular slices. Blinded to each other, two observers analysed all LGE images (AB and MS). Blinded to clinical data, the analyses were reviewed and finalized by a third observer with 10 years of CMR experience (PLvM). A LGE lesion was classified as ischemic if subendocardial or transmural and respecting the perfusion territory of a coronary artery [12], otherwise, the lesion was considered to be non-ischemic. Hinge-point fibrosis was not included, and these patients were categorised as patients without LGE lesion. A semi-automatic toll marking LGE lesions with increased myocardial signal intensity 5SD above remote myocardium was used to quantify lesion mass. Myocardial mass and mass of myocardial LGE lesions were determined from end-diastolic volumes applying a density of 1.05 g mL^−1^. On native and post-contrast T_1_-maps endocardial and epicardial borders were traced semi-automatically, and the average ECV was calculated from areas outside LGE lesions [13]. For determination of the ECV within a LGE lesion, care was taken to exclude any myocardium without LGE in the segment. Myocardial perfusion scans before and after adenosine infusion were visually inspected for perfusion defects. Using a five-point linear fit model of signal intensity vs. time myocardial perfusion up-slopes were calculated and normalized to the arterial input function. Regions with infarcts, sub-endocardial perfusion defects and dark-rim artefacts were excluded. Myocardial perfusion reserve index (MPRI) was defined as the ratio between normalized stress and rest perfusion [12].

### Statistical analysis

For association analysis, patients with both ischemic and non-ischemic LGE lesions were included in the group with ischemic lesions. Quantitative variables were analysed for normal distribution and are presented as mean and standard deviation (SD) or median and interquartile range (IQR) as appropriate. Categorical variables are presented as absolute values and percentages. A one-way analysis of variance (ANOVA) was applied for the comparison of continuous variables with a normal distribution. If an unequal variance was seen either visually or by a significant Levene’s test, Welch’s ANOVA was applied. Non-normal distributed continuous variables were compared using a Kruskal–Wallis test. Categorical variables were compared using a Chi-squared test. If an ANOVA was significant additional analyses were performed with the comparison of DM2 patients without LGE lesions to patients with non-ischemic LGE lesions using either the Student’s T-test, the Mann–Whitney U test, Fisher’s exact test or a Chi-squared test as appropriate. For all tests, a two-tailed P-value < 0.05 was considered statistically significant. All calculations were made in SAS Enterprise Guide *v*. 7.15 (SAS Institute inc., Cary, NC, USA).

### Results

296 T2DM patients and 25 sex-matched controls were included. Twenty-five T2DM patients did not complete the CMR scan, and in 6 patients the scan was performed without gadolinium contrast. In one patient, the LGE images could not be analysed because of poor image quality leaving 264 T2DM patients for final analysis. None of the 25 control subjects had any LGE lesions. In patients with T2DM, 207 (78.4%) did not have LGE lesions; 29 (11.0%) had ischemic LGE lesions only; 25 (9.5%) had non-ischemic LGE lesions only, and 3 (1.1%) patients had both an ischemic and a non-ischemic lesion. Six T2DM patients (2.3%) with ischemic LGE lesions had no prior history of ischemic heart disease. None of the patients without LGE lesions or with non-ischemic lesions had any hypoperfused areas on the adenosine stress perfusion scan indicative of significant coronary artery disease.

**Fig. 1:**
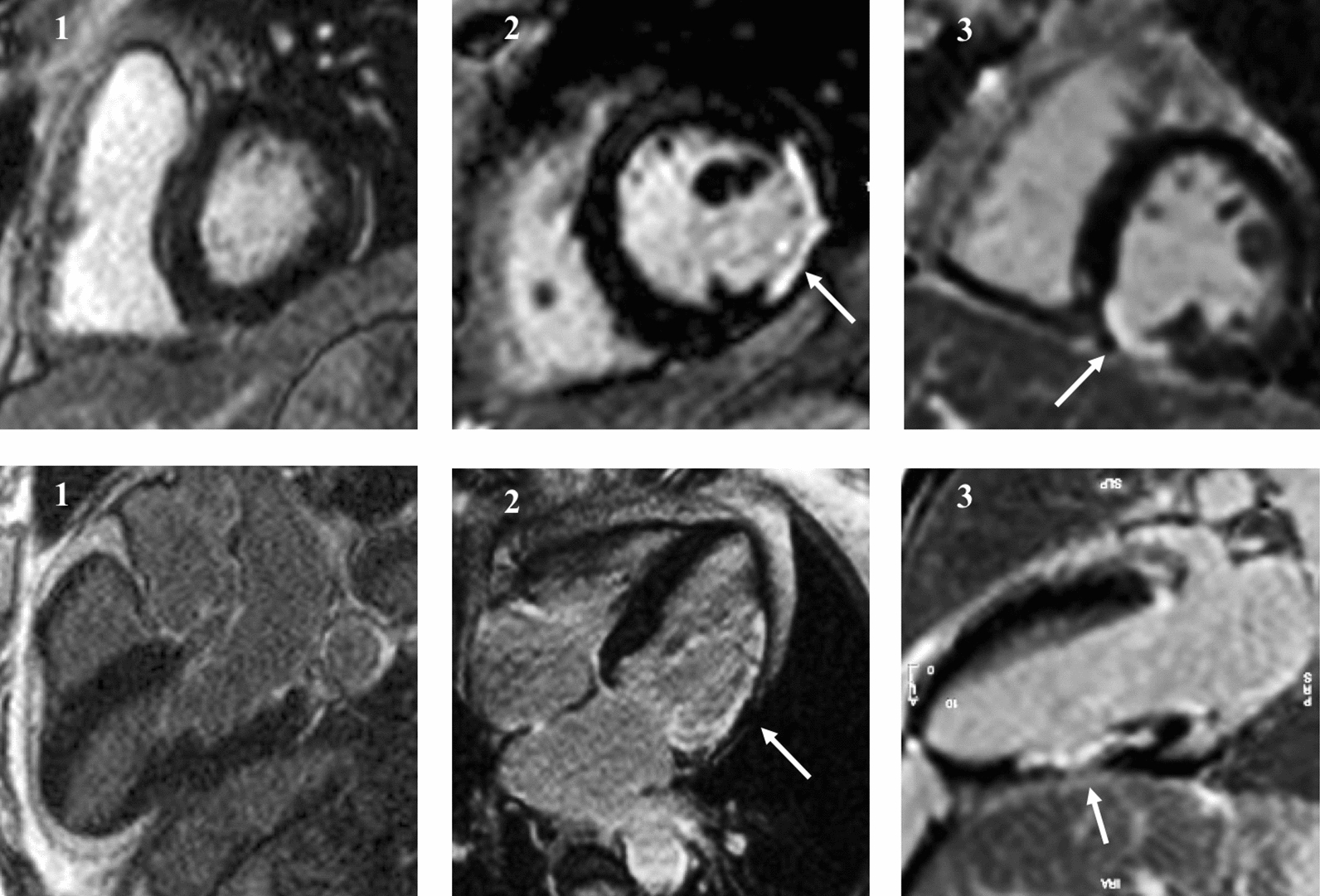
One normal subject [1] and patients with typical ischemic late gadolinium enhancement (LGE) lesions from previous myocardial infarcts [2, 3]. LV short-axis and long-axis images from the same patients. Normal myocardium appears black, whereas fibrotic areas appear white (white arrows). Ischemic lesions subendocardial/transmural, and in the area of ischemic LGE, there is thinning of the myocardium

**Fig. 2:**
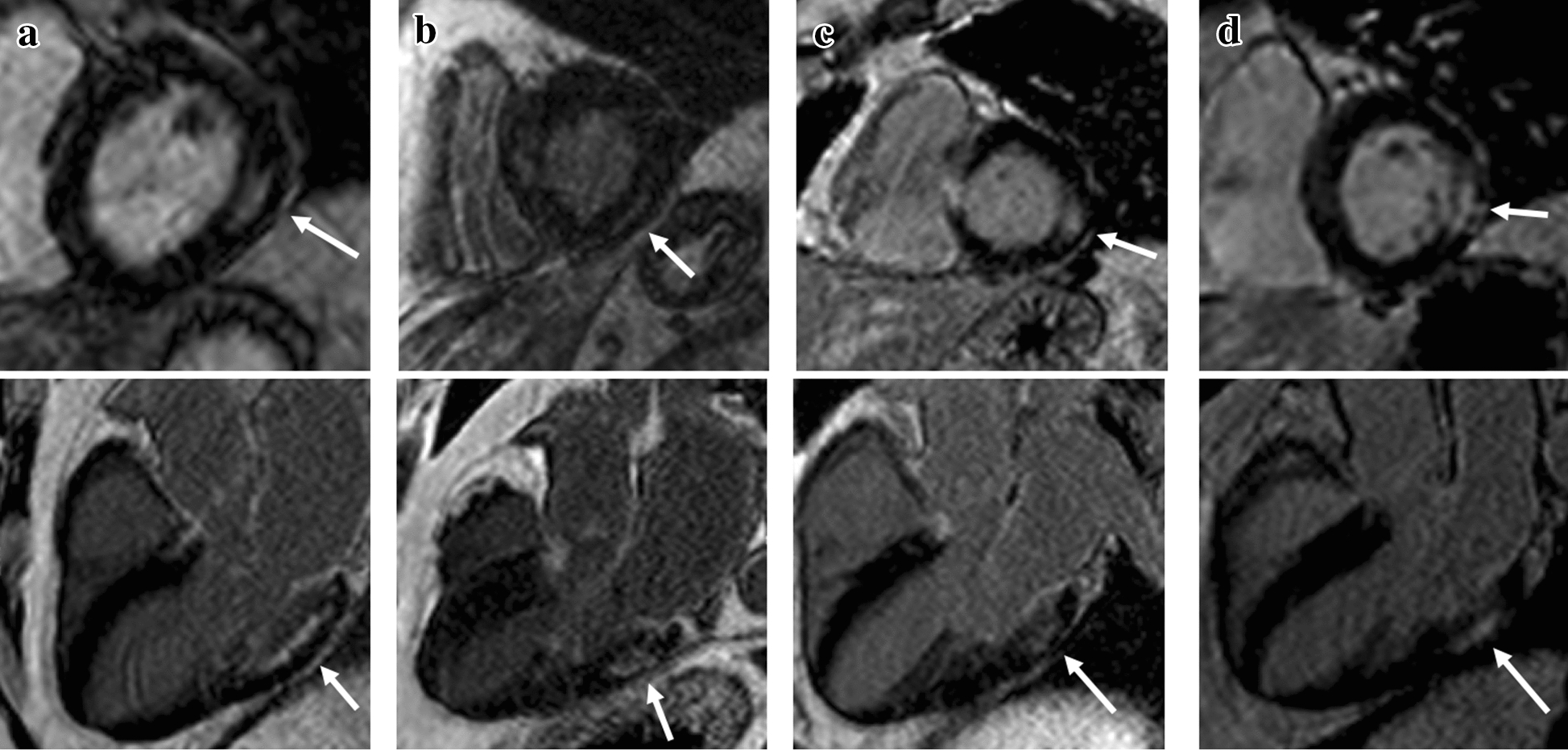
Four DM2 patients (**a**–**d**) with typical non-ischemic late gadolinium hyper-enhancement (LGE) lesions with LV short-axis and long-axis images. Non-ischemic lesions are located mid-myocardial, basal and lateral or inferolateral. In segments with non-ischemic LGE lesions, the myocardium remains thick

In all 28 patients with non-ischemic LGE lesions, these were located mid-myocardial in the basal lateral or the basal inferolateral part of the LV myocardium (Figs. 1, 2). Two patients had two non-ischemic LGE lesions, with the additional lesion located in the middle part of the interventricular septum. Non-ischemic LGE lesions appeared grey compared to ischemic LGE lesions that were well-demarcated and bright white (Figs. 1, 2). Myocardial segments of patients with ischemic lesions were to a variable degree thinned with hypo- or akinesia on cine images, whereas myocardial segments with non-ischemic LGE lesions were thick and did not present abnormal wall motion. The median mass of a non-ischemic lesion was smaller than that of an ischemic lesion (2.8 (IQR1.5–4.4) g (1.8 (IQR1.0–3.0) % of total myocardium vs. 11.7 (IQR6.9–16.1) g (7.4 (IQR5.8–9.8) % of total myocardium (P < 0.0001 for both)). At least one T_1_ mapping slice passed through the LGE lesion in 11 patients with non-ischemic and in 18 patients with ischemic LGE lesions: Without difference between non-affected remote myocardium (31.4 (IQR29.0–33.9) % vs. 33.0 (IQR31.5–35.8) %, P = 0.11), the median ECV value for a non-ischemic lesion was lower than what was seen in an ischemic lesion (40.6 IQR37.0–44.7 vs. 51.7 IQR44.9–60.8%; P = 0.004).

Patient demographics including clinical characteristics and laboratory values are presented in Table 1. In comparison with T2DM patients without LGE lesions, T2DM patients with non-ischemic LGE lesions demonstrated a trend towards higher age (P = 0.06), but though numerically higher diabetes duration there was no significant difference between any three T2DM groups. Further, patients with non-ischemic LGE lesions as compared to those without LGE had a trend towards increased systolic blood pressure (P = 0.06) but had the same diastolic blood pressure and the overall prevalence of hypertension (P = 0.11). No differences were noted between groups with respect to the number of patients in NYHA class II/III or in the number of patients treated with insulin. NT-proBNP (P = 0.02) and hs-TNT (P = 0.007) concentrations were higher in patients with non-ischemic LGE lesions than in patients without LGE.

**Table 1:** Patient characteristics

	Controls	T2DM without LGE	T2DM with ischemic LGE^a^	T2DM with non-ischemic LGE	P value^b^	P value^c^
Number	25	207	32	25	–	–
Age, years	57 IQR50–64	59 IQR50–67	64 IQR56–69.5	64 IQR57–72	0.008	0.06
Body mass index, kg/m^2^	25.1 ± 3.3	31.0 ± 4.7	31.9 ± 4.9	30.8 ± 3.7	< 0.001	0.8
Male sex (n,%)	17 (68)	140 (68)	24 (75)	23 (92)	0.08	–
Diabetes duration, years	–	12.2 ± 8.2	12.4 ± 8.0	15.4 ± 9.0	0.20	–
Systolic blood pressure, mmHg	133 ± 16	134 ± 18	142 ± 21	142 ± 14	0.04	0.06
Diastolic blood pressure, mmHg	81 ± 10	81 ± 9	82 ± 13	81 ± 9	0.98	–
Active or former smoker (n,%)	11 (44)	135 (65)	29 (91)	16 (64)	0.003	1.0
History of ischemic heart disease (n,%)	–	15 (7)	26 (81)	3 (12)	< 0.001	0.4
History of hypertension (n,%)	4 (25)	137 (66)	28 (87.5)	21 (84)	< 0.001	0.07
NYHA class II/III (n,%)	–	41 (20)	8 (25)	6 (24)	0.82	–
Insulin treatment (n,%)	–	120 (58)	18 (56)	18 (72)	0.38	–
HbA1c, mmol/mol	35 ± 3	62 ± 15	62 ± 16	64 ± 14	< 0.001	0.65
Creatinine, µmol/L	86 IQR67–89	71 IQR59–86	75 IQR65–87	74 IQR65–90	0.052	–
LDL cholesterol, mmol/L	3.0 IQR2.2–3.7	1.9 IQR1.5–2.5	1.4 IQR1.2–2.0	1.8 IQR1.3–2.8	0.0001	0.68
High–sensitive CRP, mg/L	–	2.1 IQR1.0–4.3	2.3 IQR1.0–4.0	2.5 IQR1.3–5.2	0.57	–
Pro-BNP, µmol/L	–	5.9 IQR5.9–10.1	8.0 IQR5.9–17.3	8.9 IQR5.9–19.7^a^	0.02	0.02
High-sensitive TNT, ng/L	–	13.0 IQR13.0–14.6	13.6 IQR13.0–17.6	15.6 IQR13.0–26.1^a^	0.008	0.0007
Pro-ANP, µmol/L	–	57 IQR42–90	108 IQR47–174	75.5 IQR48–127	0.01	0.1

Data presented as median and interquartile range (IQR) or as nominal values with percentages in parentheses

^a^Including three patients with both ischemic and non-ischemic lesions

^b^Comparison between all three groups as appropriate

^c^Comparison between T2DM patients with non-ischemic LGE vs. T2DM patients without LGE

The cardiac structural and functional characteristics of the four groups are presented in Table 2. T2DM patients without ischemic LGE had smaller LV volumes than controls. Further, patients with non-ischemic lesions had significantly higher LV mass than both controls and T2DM patients without LGE (P = 0.02). In comparison with controls, all T2DM groups had increased myocardial ECV, and patients with non-ischemic LGE lesions had higher ECV than patients without any LGE lesions (P = 0.01). All patients with T2DM had lower MPRI compared with controls, but we observed no significant difference in MPRI between patients with non-ischemic LGE compared to patients without LGE lesions. Compared to controls, both groups with LGE lesions had lower lateral and septal e′ and increased average E/e′. In comparison with T2DM patients without LGE, patients with non-ischemic lesions had decreased lateral e′ (P = 0.0496) and increased average E/e′ (P = 0.04) and also a higher maximal LA volume (P = 0.049).

**Table 2:** Structural and functional findings

	Controls	T2DM without LGE	T2DM with ischemic LGE^a^	T2DM with non-ischemic LGE	P-value^b^	P-value^c^
Cardiovascular magnetic resonance imaging						
LV end-diastolic volume, mL	166 ± 39	149 ± 32	172 ± 50	151 ± 27	0.03	0.8
LV end-diastolic volume/BSA, mL/m^2^	83 ± 13	70 ± 13	79 ± 18	69 ± 12	< 0.0001	0.7
LV end-systolic volume, mL	63 ± 15	54 ± 17	79 ± 34	55 ± 22	0.0005	0.9
LV end-systolic volume/BSA, mL/m^2^	31 ± 6	25 ± 8	36 ± 14	25 ± 10	< 0.0001	0.8
LV mass, g	121 ± 25	133 ± 33	163 ± 52	150 ± 34^a^	0.0003	0.02
LV mass/BSA, g/m^2^	60 ± 8	62 ± 13	75 ± 19	68 ± 13^a^	0.0007	0.03
LV ejection fraction, %	62.3 ± 3.9	64.0 ± 6.4	55.5 ± 9.0	64.5 ± 9.6	< 0.001	0.8
LA volume, mL	104 IQR86.2–115.6	91 IQR75.2–100.0	106 IQR77.9–134.3	102 IQR84.6–115.2^a^	0.002	0.049
LA volume/BSA, mL/m^2^	50 IQR47–54	41 IQR36–47	48 IQR37–59	43 IQR39–52	< 0.0001	0.15
LV ECV, %	26.1 ± 1.5	28.8 ± 2.7	32.2 ± 3.8	30.4 ± 3.1^a^	< 0.0001	0.01
LV myocardial perfusion index	1.86 ± 0.17	1.37 ± 0.31	1.18 ± 0.26	1.49 ± 0.36	< 0.0001	0.2
Echocardiography						
Lateral e′	11.8 ± 3.3	9.5 ± 2.7	9.7 ± 2.5	8.6 ± 1.8 ^a^	0.0002	0.0496
Septal e′	7.4 ± 2.3	6.8 ± 1.9	5.8 ± 1.5	6.1 ± 1.6	0.009	0.2
E/A-ratio	1.21 IQR1.0–1.67	0.90 IQR0.77–1.12	0.86 IQR0.68–1.0	0.90 IQR0.77–1.15	< 0.0001	0.8
Average E/e′	8.3 IQR7.0–11.0	8.8 IQR7.4–10.7	10.2 IQR8.0–12.8	9.9 IQR8.7–12.6^a^	0.04	0.04

Data presented as median and interquartile range (IQR) or as nominal values with percentages in parentheses

^a^Including three patients with both ischemic and non-ischemic lesions

^b^Comparison between all three groups as appropriate

^c^Comparison between T2DM patients with non-ischemic LGE vs. T2DM patients without LGE

Association of DM complications between the three groups of T2DM patients is shown in Table 3. Having any type of LGE lesion was associated with a higher prevalence of retinopathy and autonomic neuropathy. The increased prevalence of autonomic neuropathy was driven by a lowered beat-to-beat variation. Patients with non-ischemic lesions more often had retinopathy (P = 0.009) and autonomic neuropathy (P = 0.005) than patients without LGE.

**Table 3:** Diabetes complications

	T2DM without LGE	T2DM with ischemic LGE^a^	T2DM with non-ischemic LGE	P-value^b^	P-value^c^
Albuminuria (%)	72 (35)	17 (55)	8 (32)	0.09	–
Retinopathy (%)	51 (25)	8 (28)	12 (48)	0.054	0.009
Autonomic neuropathy (%)	62 (30.5)	14 (47)	13 (52)	0.03	0.005
Peripheral neuropathy (%)	78 (40)	15 (52)	12 (50)	0.34	–
Beat-to-beat variation (bpm)	11.1 IQR6.8–17.8	7.3 IQR3.4–11.9	6.5 IQR3.9–11.0	0.0003	0.0029
Beat-to-beat ≤ 6 (%)	55 (27)	14 (44)	12 (50)	0.01	0.02
Orthostatic blood pressure fall (mmHg)	12.0 IQR5–18	11.5 IQR4–19.5	13.0 IQR5–21	0.34	–
Orthostatic blood pressure fall ≥ 25 (%)	27 (13)	6 (19)	3 (12)	0.66	–

Data presented as median and interquartile range (IQR) or as nominal values with percentages in parentheses

^a^Including three patients with both ischemic and non-ischemic lesions

^b^Comparison between all three groups as appropriate

^c^Comparison between T2DM patients with non-ischemic LGE vs. T2DM patients without LGE

## Discussion

In a cross-sectional magnetic resonance imaging study on diabetic heart disease, 10.6% [28] of a 264 large cohort of T2DM patients had late gadolinium enhancement lesions that could not be explained by previous infarcts (non-ischemic LGE lesions). To the best of our knowledge, such non-ischemic LGE lesions have not been described or indeed systematically studied before in patients with diabetes.

### Comparison with literature

Previous LGE CMR studies in T2DM populations have focused on ischemic heart disease with ischemic LGE lesions from myocardial infarctions [14–16]. Of interest, we found a lower prevalence of unrecognised myocardial infarcts than in previously examined T2DM cohorts [16] This could be due to different study populations but it is also likely that due to modern treatment of risk factors for ischemic heart disease in T2DM, T2DM populations now reach higher age. Perhaps therefore the non-ischemic fibrotic lesions were more prevalent than unrecognised myocardial infarcts. Small autopsy studies from the 1970–1980s have however described localised confluent fibrotic lesions in patients, who had died from diabetic cardiomyopathy without signs of coronary sclerosis and without infarction [17, 18]. Similar to ours, the non-ischemic fibrosis in these autopsy studies was placed mid-myocardial, although unfortunately no information of their anatomical localization in the left ventricle was given. In a study by Stroz et al. cardiovascular magnetic resonance imaging including LGE was performed in a smaller T2DM population of 47, all free of known cardiovascular disease [19]. Out of the 47 patients 3 patients (4.2%) had LGE lesions but unfortunately the type of LGE lesions found was not described in the study. In this study the mean duration of diabetes at 8.4 ± 5.7 was lower than in our study, which could be a possible explanation for the lower LGE prevalence found in the study. Further, the prevalence of hypertension was similar to ours. Another study by Bizino et al. performed CMR in 47 patients with T2DM without known cardiovascular disease [20]. They reported one patient with LGE in the inferoposterior basal segment, the patient had no sign of cardiac ischemia, and hence this LGE element could be of the same nature as the ones presented in this study. However, the authors did not report if the LGE lesion was sub-endocardial, transmural or mid-wall.

Patients with non-ischemic LGE lesions had increased ECV, which has previously been shown to correlate well with the levels of diffuse interstitial myocardial fibrosis [21]. Thus, one possible explanation for our findings could be that the non-ischemic LGE lesions represent an exceedingly high local concentration of fibrosis becoming so high as to be seen with the conventional LGE technique. This could be a possible explanation for the increase in LV mass seen in the group. Compared to controls all patients with T2DM had increased LV mass consistent with previous studies [22], but LV mass was further increased in patients with non-ischemic LGE indicating that this group had more pronounced cardiac involvement. The non-ischemic LGE lesions, may also be related to microvascular complications as the lesions were statistically associated with retinopathy and autonomic neuropathy. This increased prevalence of these complications of diabetes indicates that non-ischemic LGE lesions could be a result of poorly treated DM. However, further research is needed to determine the aetiology and mechanisms leading to these lesions.

Non-ischemic LGE lesions are well-known in patients with other non-ischemic cardiomyopathies such as hypertrophic cardiomyopathy, idiopathic dilated cardiomyopathy, myocarditis, Chagas disease, sarcoidosis, Anderson-Fabry or cardiac amyloidosis [23]. In all cases, the LGE pattern differs from the quite specific pattern in patients with T2DM found in our study. Only few and smaller CMR studies have been done in other patients populations known to develop heart failure. But LGE patterns have been described in patients with uraemia (of whom some also had DM) [24, 25], hypertensive cardiomyopathy [26], aortic stenosis [26], atrial fibrillation [27] and exercise-induced hypertrophy [28]. In none of the studies a typical pattern of LGE was described. Further, unfortunately most of these studies did not report prevalence of diabetes. In our study, it was not possible to isolate the effect of DM from that of hypertension often seen in T2DM. The prevalence of hypertension was not higher in patients with non-ischemic LGE lesions, however, there was a trend towards higher measured systolic blood pressure, but non-ischemic LGE lesions could be seen despite normal blood pressure.

In other cardiomyopathies, the finding of non-ischemic LGE lesions is associated with prognosis, and if such is the case in T2DM this now has to be established. Non-ischemic LGE lesions have in other patient groups been correlated to heart failure, arrhythmias, and sudden cardiac death [27, 29, 30]. The non-ischemic LGE lesions found in our study were related to parameters associated with impaired diastolic function, in itself an important prognostic factor. Further, non-ischemic LGE lesions were related to increments in prognostic important biomarkers. Thus, increased NT-pro-BNP relates to cardiac remodeling [31] and hs-TNT with cardiac myocyte damage [32] and, hence, these findings suggest that the non-ischemic LGE is associated with the adverse structural remodelling shown in the group.

## Limitations

Our work have some limitations, first the study was cross-sectional and we can only infer causality and speculate on the causes of and consequences of having non-ischemic LGE lesion from the result on other patient groups. Also we excluded patients with nephropathy (eGFR < 30 mL/min/1.73m^2^) since we used gadolinium contrast for the CMR scans. An overlap exists between diabetic nephropathy and albuminuria and having excluded this patient group we may have missed an association between non-ischemic LGE lesions and albuminuria. Further, we have not performed biopsies, and thus we can only speculate on the aetiology of the lesions based on statistical associations. In this study we did not perform high resolution LGE or feature tracking, both newer and emerging techniques that could possibly have helped phenotyping our cohort even more [33, 34]. However, as LGE of the non-ischemic type was generally smaller than the ischemic lesions we speculate that there would not have been detectable regional strain differences in the none-ischemic lesion areas.

## Conclusions

A localised non-ischemic LGE lesion pattern situated mid-myocardial, basal lateral or basal inferolateral is associated with a severe variant of the diabetic cardiac phenotype with increased myocardial mass, increased myocardial extracellular volume, impaired diastolic parameters, increased biomarkers and prevalent complications of diabetes. Further studies correlating magnetic resonance imaging findings with prognosis and pathology are now needed to explain these lesions and their importance for prognosis.

## Data Availability

Beginning 3 months and ending 5 years from publication, data will be shared with researchers who provide a methodologically sound proposal and who get all the appropriate approvals. Individual participant data that underlie the results and the study protocol can be shared. Proposals should be directed to the corresponding author.

## Abbreviations

CMR: Cardiovascular magnetic resonance imaging
ECG: Electrocardiogram
ECV: Extracellular volume
DM: Diabetes
IQR: Interquartile range
LGE: Late gadolinium enhancement
MPRI: Myocardial perfusion reserve index
SD: Standard deviation
T2DM: Type 2 diabetes
